# Tuning and Freezing Disorder in Photonic Crystals using Percolation Lithography

**DOI:** 10.1038/srep19542

**Published:** 2016-01-21

**Authors:** Ian B. Burgess, Navid Abedzadeh, Theresa M. Kay, Anna V. Shneidman, Derek J. Cranshaw, Marko Lončar, Joanna Aizenberg

**Affiliations:** 1 Leslie Dan Faculty of Pharmacy, University of Toronto, Toronto, Ontario Canada; 2 Wyss Institute for Biologically Inspired Engineering, Harvard University, Cambridge, MA, USA; 3 John A. Paulson School of Engineering and Applied Sciences, Harvard University, Cambridge, MA, USA; 4 Department of Chemistry and Chemical Biology, Harvard University, Cambridge, MA, USA; 5 Kavli Institute for Bionano Sciences and Technology, Harvard University, Cambridge, MA, USA

## Abstract

Although common in biological systems, synthetic self-assembly routes to complex 3D photonic structures with tailored degrees of disorder remain elusive. Here we show how liquids can be used to finely control disorder in porous 3D photonic crystals, leading to complex and hierarchical geometries. In these optofluidic crystals, dynamically tunable disorder is superimposed onto the periodic optical structure through partial wetting or evaporation. In both cases, macroscopic symmetry breaking is driven by subtle sub-wavelength variations in the pore geometry. These variations direct site-selective infiltration of liquids through capillary interactions. Incorporating cross-linkable resins into our liquids, we developed methods to freeze in place the filling patterns at arbitrary degrees of partial wetting and intermediate stages of drying. These percolation lithography techniques produced permanent photonic structures with adjustable disorder. By coupling strong changes in optical properties to subtle differences in fluid behavior, optofluidic crystals may also prove useful in rapid analysis of liquids.

Several decades of studying periodic photonic systems, from thin films to 3D crystals, have produced numerous significant technological advances^1^,^2^,^3^,^4^. This was made possible by developments in nanoscale manufacturing^5^,^6^,^7^,^8^,^9^,^10^,^11^, including the large-scale production of periodic photonic structures by self-assembly methods^8^,^9^,^10^,^11^. More recently the study of aperiodic photonic structures, ranging from small defects in periodic lattices^12^,^13^,^14^ to disordered and quasi-periodic structures^15^,^16^,^17^,^18^,^19^, has led to the prediction and discovery of a much more diverse range of optical effects, with wide-ranging applications from random lasers^20^ to structurally colored materials with precisely controllable wavelength and angular dependence of scattering^21^. While arbitrary structures with designer symmetries, defect distribution and optical properties can be fabricated in 1D and 2D by planar nanofabrication techniques^22^,^23^,^24^, our ability to fabricate precisely controlled aperiodic, disordered or partially disordered 3D photonic structures remains limited. Furthermore, even in 1D and 2D, disordered patterns must be individually written, and robust methods for dynamically tuning and randomizing disorder in a single photonic structure do not exist.

In contrast, various organisms, including butterflies, beetles and birds, have evolved complex 3D periodic and aperiodic morphologies with varying degrees of disorder, displaying multifaceted optical properties^25^,^26^,^27^,^28^,^29^. These structures are all produced by self-assembly processes, often exploiting simple interfacial chemistry and physics under controlled conditions to make and break symmetries in tailored ways^30^. Inspired by these biological examples, we studied how simple capillary phenomena driven by interfacial physics can be used to create complex and hierarchical 3D photonic structures with a tunable degree of disorder. Guided by a theoretical model, we demonstrate how the capillarity-driven partial imbibition of a liquid into (Fig. 1A) or drainage of a liquid from (Fig. 1B) a porous 3D photonic crystal creates a continuum of complex and hierarchical 3D photonic structures with a precisely controlled degree of disorder. This is enabled by subtle variations in the morphology of the photonic crystal lattice (on scales much smaller than wavelength of light^31^) that lead to specific, transient percolation patterns of randomly distributed, partially filled pores. These modifications have significant effects on the angular and wavelength dependence of scattering, providing unique optical signatures to the transient structures. By incorporating cross-linkable resins into the liquids, thus allowing filling patterns to be frozen at arbitrary stages of incomplete wetting or drying, this percolation lithography process produces permanent 3D photonic structures with specific levels of disorder. This optofluidic approach, broadly applicable to many types of photonic backbones, may provide an experimentally accessible route to many optical phenomena associated with controlled disorder that were previously only explorable theoretically.

## Results and Discussion

**Figure 1:**
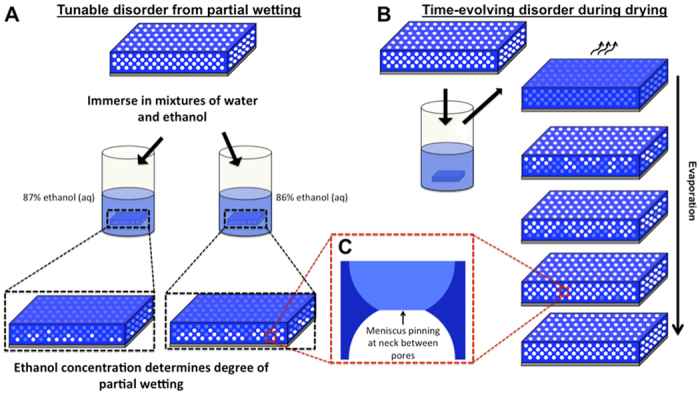
Tuning disorder in inverse-opal films (IOFs) via partial wetting and drying. (**A**) Immersing IOFs in mixtures of water and ethanol, whose proportions place the mixture near the structure’s wetting threshold^31^, creates disordered partial wetting states in which degree of wetting can be tuned with small adjustments in the ethanol content. Silica matrix is represented with dark blue color, air pores with white color, and holes filled with fluid with light-blue color. (**B**) A time evolution of disordered partial filling patterns occurs as liquid evaporates from an initially fully infiltrated IOF. (**C**) Partial filling patterns occurring in both processes are directed by variability in the geometry of the necks between adjacent pores, leading them to pin menisci with varying strengths.

The 3D photonic crystals used in this work were inverse-opal films (IOFs) made of silica, and were fabricated on silicon substrates using an evaporative co-assembly method described previously^32^. IOFs produced by this technique contain regularly spaced spherical voids (*d* ~ 300 nm) arranged in a face-centered-cubic lattice with a single crystallographic orientation and very low defect density across centimeter scales. Neighboring voids are connected by small circular openings – *necks* – that have a highly re-entrant geometry, which encourages contact-line pinning as a liquid menisci pass through them^31^,^33^ (Fig. 1C). At the necks, there is a significant free-energy barrier for the contact line to overcome during imbibition and drainage^31^. While in an ideal photonic crystal, the pore geometry is exactly the same at all lattice sites, small variation in the pore and neck geometry is always observable in real, self-assembled IOFs^31^. In contrast to previous work, where we have shown how this near-ideal geometry results in wetting transitions (from complete imbibition to complete non-wetting) that occur over a very small range of contact angles (~3°)^31^, we focus here on the structure’s imperfections and how they can be used to direct large-scale patterns in imbibition and drainage. The scale of this broken symmetry is much smaller than visible wavelengths and therefore it has little direct effect on the IOFs optical properties. However, variation in the neck geometry will translate into subtle differences in the amplitude of the free energy barriers between different lattice sites, and thus can selectively direct the imbibition^31^ or drainage^34^,^35^ of liquids. In this way, irregular macroscopic hierarchical wetting patterns can be superimposed onto the underlying periodic photonic crystal.

### Theoretical Analysis of Filling Patterns Produced by Partial Imbibition and Drainage

**Figure 2:**
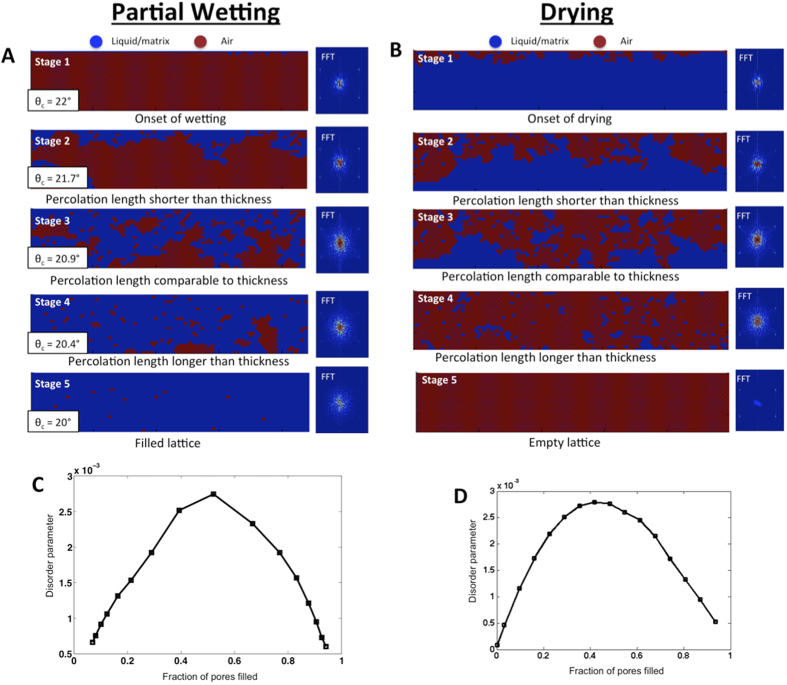
Simulated evolution of disorder in inverse-opal films (IOFs) due to partial wetting and drying. (**A**,**B**) Percolation simulations of 2D IOFs showing the progression of defect patterns occurring during partial wetting as a function of (**A**) the liquid’s intrinsic contact angle, *θ*_c_^31^, and (**B**) drying as it evolves in time. Fourier transforms (FTs) of the refractive-index map are shown to the right of each image. The density of the FT outside of the vertical axis expands and contracts with the degree of disorder. (**C,D**) Disorder, quantified from the lattice FTs as the fraction of spectral density in the first Brillouin zone outside of the vertical axis, plotted as a function of the overall filling fraction, for partial wetting (**C**) and drying (**D**). This region is chosen to separate the contributions of lattice disorder from the FT density associated with reciprocal lattice sites, the zero-spatial-frequency component at the origin, and the contributions along the vertical axis coming from the top and bottom interfaces of the film. Maximum disorder occurs in both cases when half of the pores are filled.

Simulated refractive-index maps (left) and their Fourier Transforms (FTs, right) derived from our simulations of partial wetting and drying on 2D IOFs at five characteristic stages are shown in Fig. 2A,B, respectively (see Methods for simulation details). Figure S1 (Supplementary Information) shows the evolution of the fraction of pores filled as a function of depth. The evolution of partial filling patterns in partial wetting and drying are very similar in nature, except that the locations of liquid and air are interchanged. The process can be described as the propagation of the invading species – i.e. liquid in the case of the wetting or air in the case of drying – into the IOF, replacing the receding species – i.e. air in the case of the wetting or liquid in the case of drying.

In both cases, pattern progressions can be divided into five characteristic stages, which are distinguished by the relationship between the percolation length and the film thickness. Stage 1 marks the onset of pore filling (partial wetting) or pore emptying (drying) on an initially ordered lattice. At Stage 2, the wetting/drying front has penetrated into the lattice, but the percolation length is shorter than the total thickness of the film and the deepest layers remain relatively unperturbed. When the percolation length is shorter than or comparable to the film thickness (Stages 1–3), the fraction of pores filled by the invading species decreases with depth, reflecting the decreasing probability of connected paths to extend to pores at larger depths. We define Stage 3 as the point where the percolation length is comparable to the thickness, the lattice is half-filled and the invading species becomes the majority in both wetting and drying. At Stage 4, the percolation length is larger than the thickness and the receding species become increasingly isolated defects that are homogeneously distributed in the film. Once the percolation length has grown well past the thickness of the film (eventually crossing the percolation threshold where the percolation length, *L *→∞), the probability of finding connected paths to the surface no longer varies with depth and the filling fraction becomes homogeneous. Stage 5 marks the completion of wetting or drying and recovery of a completely liquid-filled lattice or defect-free, air-filled photonic crystal, both of which are ordered structures.

In both partial wetting and drying, the degree of disorder initially increases as the percolation progresses from an ordered state at the outset and reaches a maximum at the point of half filling (Stage 3, Fig. 2C,D). Once the percolation progresses past this point, disorder decreases, as the invading species become the majority and the receding species become the defect centers. Figure 2C,D plots a measure of disorder in the lattice as a function of the overall filling percentage, showing its maximization at 50% filling in both scenarios.

### Characterization of the Optical Response

**Figure 3:**
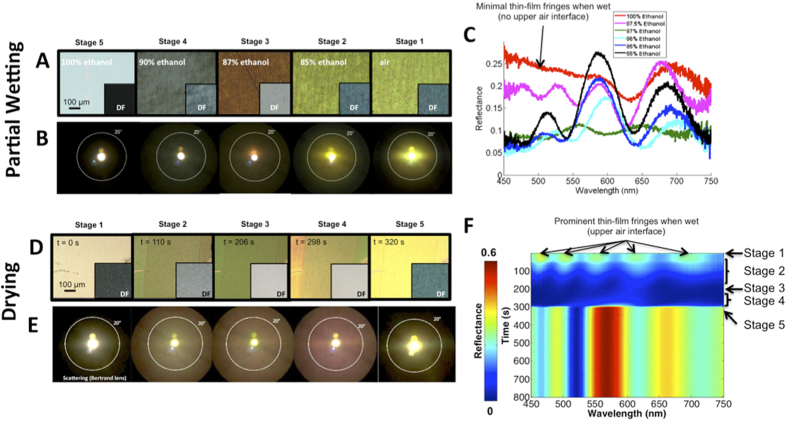
Experimental evolution of color and scattering during partial wetting and drying. (**A**) Bright-field and dark field (insets) images of a DEC-functionalized IOF (9 layers) immersed in different ethanol-water mixtures. (**B**) Corresponding Bertrand lens images, showing angular distribution of scattering. (**C**) Reflectance spectra at normal incidence of an IOF (9 layers) immersed in various ethanol-water mixtures, showing the effects of partial wetting. (**D**) Time lapse bright field and dark field (insets) images of an IOF (9-layers) as dodecane evaporates from the pores. (**E**) Corresponding Bertrand lens images, showing angular distribution of scattering. (**F**) Time evolution of reflectance at normal incidence for an IOF (9 layers).

To experimentally validate these concepts, we tuned the degree of partial wetting by immersing IOFs, uniformly functionalized with n-decyl trichlorosilane (DEC) to render their pores hydrophobic, in different mixtures of water and ethanol (Fig. 3A–C). Adjusting the ratio of water and ethanol in the mixtures allowed us to create a continuum of *θ*_c_ values in the pores^31^. We experimentally observed the time evolution of disorder during drying after filling IOFs with thin films of liquid alkanes (decane, undecane, dodecane) (Fig. 3D–F). Specific alkanes were chosen based on their volatility, allowing us to set the ideal drying timescale for the different experiments. Figure 3A,D show bright-field and dark-field images of the IOFs having 9 close-packed layers in thickness for the five stages described in Fig. 2A,B and discussed above. These images are accompanied by images taken through a Bertrand lens^36^ that map the angular distribution of scattering at each stage (Fig. 3B,E). Supplementary Movies M1,M2–M3 show the complete time evolution of the dodecane drying experiments in bright-field (M1), dark-field (M2) and imaged through the Bertrand lens (M3). Reflection spectra (normal incidence) for partial wetting are shown for several ethanol-water mixtures that cover the five stages (Fig. 3C). The time-resolved reflection spectrum for dodecane drying is shown in Fig. 3F.

As partial-filling patterns produced in wetting and drying (compare Fig. 2A,B) are very similar in nature, there are also many commonalities in their optical signatures (compare Fig. 3A–C and 3D–F). In both scenarios, the spectral evolution has two main characteristics that occur simultaneously. First, the degree of diffuse scattering increases and then decreases after reaching a maximum (Stage 3), following the expected evolution in the degree of disorder (Fig. 2C,D) as the percolation processes evolve. Increased diffuse scattering results in a decrease in brightness of bright-field images (Fig. 3A,D), an increase in brightness of dark-field images (Fig. 3A,D, insets), a broadening of the bright area in the Bertrand-lens images (Fig. 3B,E), and a decrease in the total reflectance at normal incidence evident in the spectra (Fig. 3C,F). Second, the qualitative characteristics of the spectra (and the associated color) are transitioning from having a Bragg resonance when the pores are predominantly air-filled (wetting: Stage 1; drying: Stage 5) to having thin-film characteristics (wetting: Stage 5; drying: Stage 1), when the pores are predominantly liquid-filled, reflecting the relatively close refractive-index match between the liquid and the matrix.

The completely filled thin-film stage reveals the most important difference between the spectral response in partial wetting and drying experiments, which arises from the region directly above the structure being liquid at all stages in our wetting experiments and being air at all stages in drying experiments. This prominent upper interface in the drying experiments leads to enhanced appearance of thin-film fringes in the completely wet (beginning) stage of drying experiments (Fig. 3F) compared to the completely wet IOFs in wetting experiments (Fig. 3C). While barely noticeable in the completely wet state, thin film fringes are more prominent in Stage 4 (e.g. 87.5% ethanol in Fig. 3C). This effect arises because the contrast in average refractive index between the IOF and the liquid above becomes more prominent as the density of roughly uniformly distributed empty pores increases. The presence of a greater refractive-index contrast at the top surface in drying also leads to the stronger overall reflectance compared to partial wetting at all stages.

**Figure 4:**
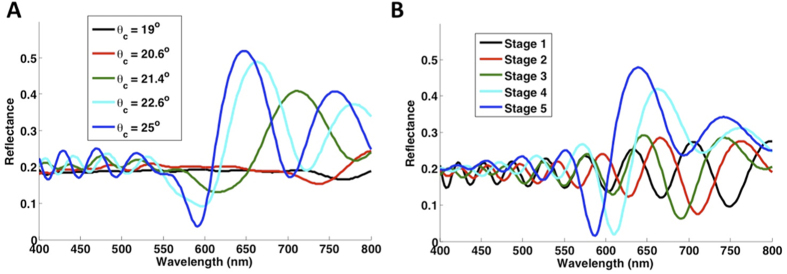
1D simulations of the spectral percolation response. Simulated reflectance calculated using the transfer matrix method for 1D refractive-index profiles generated by percolation simulations (Fig. 2A,B) for partial wetting (**A**) and drying (**B**) in a 2D silica IOF on a silicon substrate. 1D refractive-index profiles were generated by averaging the dielectric constant from the 2D refractive-index profiles across the horizontal dimension. Redshifting of spectral features occurs with increased filling in both sets of simulations, as is observed experimentally. However, the 1D simulations do not capture the primary effect of transverse disorder: the dramatic drop and recovery in the total reflectance that occurs as percolation progresses.

The thin-film character of the reflectance spectrum is maintained for much of the majority-liquid-filled stage (wetting: Stage 4; drying: Stage 2) between complete liquid filling and the point of maximum scattering. Likewise, a prominent peak corresponding to the Bragg resonance is also observed for much of the majority-air-filled stage (wetting: Stage 2; drying: Stage 4) between maximum scattering and complete air filling. However, side fringes are significantly suppressed with increased liquid filling during this stage, a consequence of transverse disorder. In both transition stages for wetting and drying experiments, redshifting of spectral features occurs with increased liquid filling (see Fig. 3C,F). This trend reflects the increase in the average optical path length between pores as more are filled with the higher-index liquid. To confirm this explanation for the observed shifts, we conducted 1D optical reflectance simulations (transfer matrix method) on the refractive index profiles generated by our percolation simulation after averaging the dielectric constant across all transverse coordinates at each depth. This simulation (Fig. 4), which serves to remove the effects of transverse disorder, reproduces much of the qualitative behavior of the experimentally observed spectral evolution, including the transition from thin film to Bragg resonance, the redshifting of spectral features with increased filling, and the changes in intensity of interference fringes between Stage 4 and Stage 5 of partial wetting. However, it does not capture the sharp decrease in the overall reflectance intensity at intermediate filling stages, which are a result of transverse disorder.

**Figure 5:**
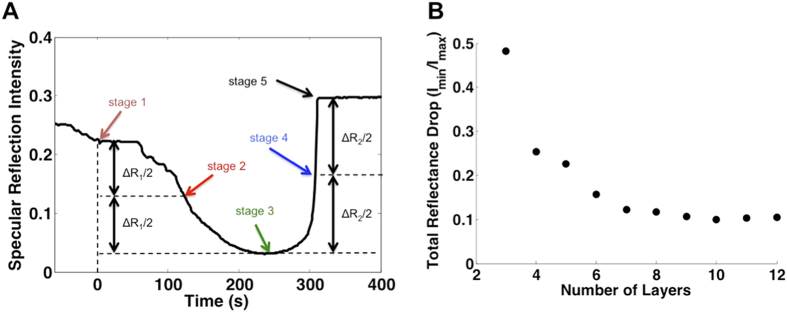
Thickness dependence of spectral evolution during drying. (**A**) Time evolution of total reflectance between 450 nm and 750 nm (*t* = 0 denotes the disappearance of excess liquid above the IOF) during drying of dodecane from a 12-layer IOF. Spectral signatures were compared at five different stages defined by the total reflectance: Stage 1: disappearance of the over-layer and start of pore-emptying; Stage 2: Total reflectance decreased to halfway between Stage 1 and the minimum reflectance; Stage 3: Point of minimum reflectance; Stage 4: Total reflectance recovered halfway from the minimum value toward the end (dry) value; Stage 5: Drying completed. (**B**) Thickness dependence of the total reflectance change, quantified as the ratio of minimum (Stage 3) and maximum (Stage 5) reflectance.

Changing the total thickness of the IOF had two notable consequences on the way its optical properties evolve during percolation. First, the strength of transverse scattering and the suppression of reflectance when disorder is at its maximum (Stage 3) increase with film thickness (Fig. 5). Second, the qualitative transition of the reflectance spectrum from thin-film character to photonic-crystal character (the appearance of a Bragg resonance) occurs after the point of minimum reflectance (maximum disorder) for the thinnest samples (*h* < ~6 layers), while it occurs before this point for thicker samples (see Fig. S2 for time-resolved reflection spectra of dodecane evaporation from IOFs with 3–11 layers).

**Figure 6:**
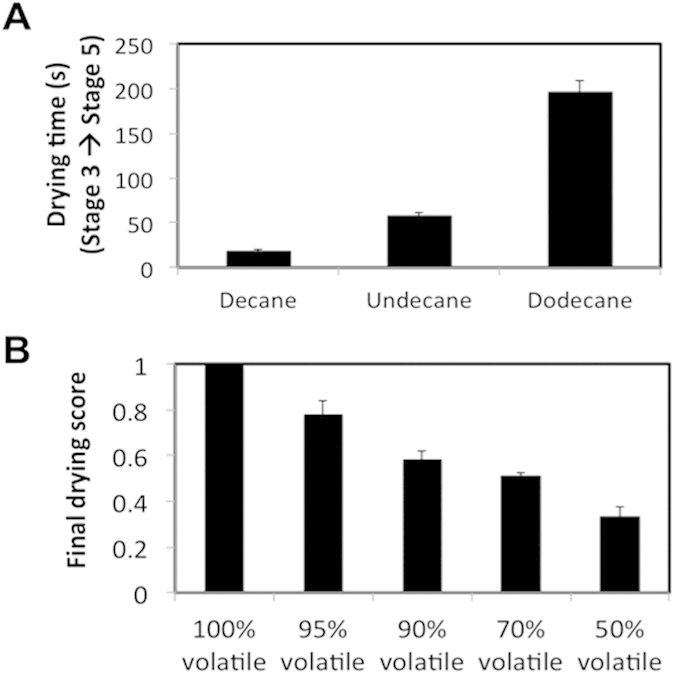
A colorimetric test for volatility profile in liquids and liquid mixtures. (**A**) Drying time, measured between Stage 3 and Stage 5, for decane, undecane and dodecane evaporating from a 9-layer IOF. (**B**) Final drying scores, quantifying the stage of progression at which the optical signature stopped changing (indicating complete evaporation of the volatile component) and plotted as a function of the initial volatile fraction, taken for dodecane-hexadecane mixtures. The scoring system used to quantify the progression of drying on a scale from 0 (no pores emptied) to 1 (completely dry) using total reflectance is defined as follows: Scores at each of the five stages (as defined in Fig. 5) are fixed: 0 for Stage 1; 0.25 for Stage 2; 0.5 for Stage 3 (minimum reflectance); 0.75 for Stage 4; 1 for Stage 5, and intermediate values interpolated using relative total reflectance intensity. For example, reflectance that has passed the minimum and then recovered 4/5 of its dry value would be given a score of 0.9 (i.e. 4/5 of the way between 0.5 and 1). All error bars represent standard error.

While the time taken to reach each stage varies greatly between the liquids and depends on volatility, the spectral signatures themselves showed relatively little difference, reflecting only the extent that the pores have dried (Fig. S3). Therefore, optofluidic crystals can be used for rapid volatility analysis of liquids or liquid mixtures. For pure substances, the time between distinct optical signatures provides a reproducible measure of volatility. Figure 6A shows the time between Stage 3 (minimum reflectance) and Stage 5 (complete drying) for three experiments on a 9-layer IOF in each of decane, undecane, and dodecane, illustrating the reproducibility of the time signature (less than 10% variability in all cases). In mixtures of liquids with different volatility, the time evolution of the optical signature also provides information on their relative concentrations. This is illustrated in Figs 6B and S4 for mixtures of a volatile (dodecane) and a non-volatile (hexadecane) component. Drying in these mixtures proceeds until the volatile component evaporates, leaving the non-volatile component behind. We used a scoring system to quantify the extent of drying at which the mixtures reached equilibrium (after the volatile component had completely evaporated) using changes in the total reflectance, running on a scale from 0 (completely filled) to 1 (completely dry). The scoring system is defined as follows: Scores at each of the five stages (as defined in Fig. 5) are fixed: 0 for Stage 1; 0.25 for Stage 2; 0.5 for Stage 3 (minimum reflectance); 0.75 for Stage 4; 1 for Stage 5, and intermediate values interpolated using relative total reflectance intensity. For example, reflectance that has passed the minimum and then recovered 4/5 of its dry value would be given a score of 0.9 (i.e. 4/5 of the way between 0.5 and 1). The uncertainty in the thickness of the thin liquid layer above the IOF at the start of the drying experiment made sensitivity to composition increase in samples with higher volatile fraction.

### Percolation Lithography

**Figure 7:**
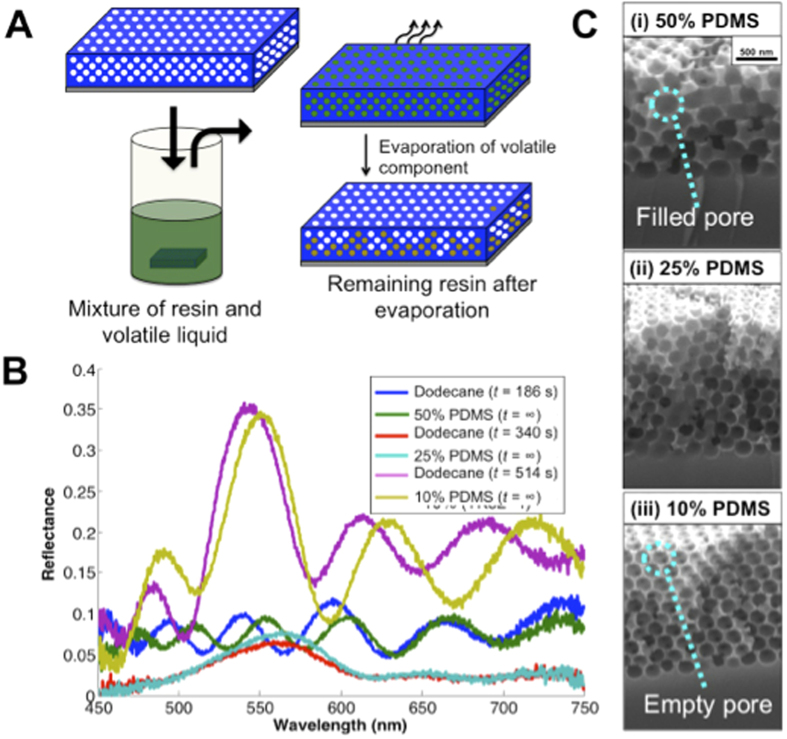
Freezing intermediate stages of drying. (**A**) Schematic showing an IOF wetted in a mixture of volatile liquid and curable resin. Evaporation-driven percolation proceeds until only the non-volatile resin remains, at which time the resin is cured. (**B**) Reflection spectra of frozen partially PDMS-filled IOFs (9 layers) made from evaporating decane-PDMS mixtures with different starting concentrations (i, 50% PDMS, ii, 25% PDMS, iii, 10% PDMS). Spectra from time-points from a drying experiment with pure dodecane are shown for comparison (i, *t* = 186 s, ii, *t* = 340 s, iii, *t* = 514 s). (**C**) SEM images of cross-sections of the partially PDMS-filled IOFs from (**B**).

Replacing the non-volatile component with a curable-resin, we can permanently freeze in place the defect pattern at arbitrary intermediate stages of the drying event (see schematic in Fig. 7A). This process, which we term ‘percolation lithography’, enables the fabrication of photonic structures with precisely controlled and highly tunable levels of disorder and their corresponding reflection spectra. Specifically, we filled IOFs with mixtures of decane and polydimethylsiloxane (PDMS) resin. After allowing drying to proceed until the decane component evaporated, we thermally cured the PDMS to solidify the structure in place. The volume fraction of non-volatile PDMS resin determined the stage of percolation in which the structure would freeze and thus its optical properties. Figure 7B reproduces the optical signatures of IOFs during different time-points of dodecane drying (approximating Stages 2, 3 and 4) replicated in permanent partially disordered IOFs created from different starting PDMS concentrations. Scanning electron micrographs (SEM) of cross-sections of these IOFs, showing the permanent partially filling structures are shown in Fig. 7C. The initial volume fractions of PDMS were significantly lower than the final fraction of pores filled (e.g. 25% PDMS produced roughly 50% filling), as is evident from the images in Fig. 7C. This disparity reflects the slight excess of liquid present as a thin film that remains on top of the structure at the start of evaporation^37^,^38^.

**Figure 8:**
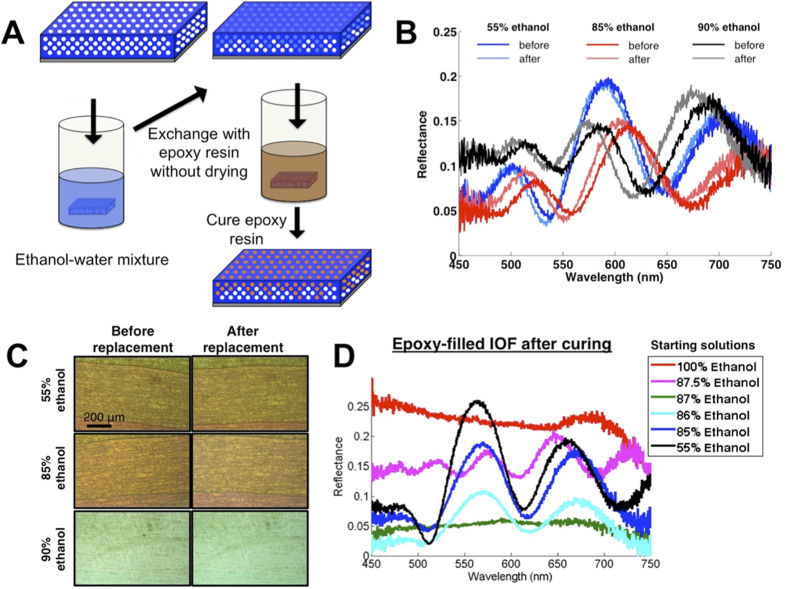
Freezing partial wetting patterns. (**A**) Schematic showing the procedure. An IOF is immersed in an ethanol-water mixture after which exchange with OG 142 epoxy resin is performed, followed by UV curing. (**B**,**C**) Spectra (**B**) and optical microscope images (**C**) of three partial wetting patterns in a DEC-functionalized IOF (9 layers) induced by immersing the dry structure into three different ethanol-water mixtures (“before”) and maintained after the ethanol-water mixtures are exchanged with pure water without drying. (**D**) Spectra of IOFs (9-layers) taken after the ethanol-water mixtures shown in Fig. 3C have been exchanged with epoxy resin and then cured.

An analogous percolation lithography approach to permanently freeze in place disordered photonic structures produced by partial wetting is shown schematically in Fig. 8A. This method exploited the fact that wetting in IOFs is limited by metastable pinning at the necks and not a global free-energy minimum^31^. As a result, we can exchange a liquid that has partially wet an IOF with a second miscible liquid without changing the partial wetting profile as long as the intrinsic contact angle does not decrease during the mixing process. Figure 8B,C shows a proof of this concept. We wetted an IOF (9 layers in total thickness) in various mixtures of ethanol in water that produced different degrees of wetting (55%, 85% and 90% ethanol) and then purged them in water without drying first. This resulted in no significant changes to either the reflection spectrum (Fig. 8B) or the color of the films (Fig. 8C), aside from very small shifts due to the slight refractive-index difference between the ethanol-water mixtures and pure water.

We exploited this effect to freeze partial wetting patterns produced in different ethanol-water mixtures, by exchanging the mixture with epoxy resin instead of water. Like water, this epoxy resin was found to not infiltrate the pores when exposed to dry DEC-functionalized IOFs, indicating a wettability to the pore surfaces that was always poorer than the starting ethanol-water mixture, the critical requirement for maintaining the filling state dictated by the first mixture. Figure 8D shows the reflection spectra of segments of an IOF (9 layers in total thickness) in ethanol-water mixtures that produce various different partial filling patterns (see Fig. 3C) after these mixtures have been replaced with epoxy and cured, illustrating that the full range of partial wetting patterns can be frozen in epoxy. Figure S5 compares the spectra for each sample in the ethanol-water mixtures and in the epoxy resin before and after curing. In contrast to the ethanol-water to water exchange (Fig. 8B,C), there is a considerable refractive difference between the epoxy (*n* = 1.58) and the ethanol-water mixture (*n* ~ 1.35) that causes the spectral features to significantly blueshift upon exchange. No shifts of spectral features were observed during curing, indicating that the epoxy remains effectively pinned in place and expected volume changes during curing are accommodated by excess epoxy resin present above the structure.

### Outlook

These results illustrate the expanded capabilities enabled in nanoscale structures that are optimized for multiple functions, in this case, correlating wetting and optics. In biological systems, such as the photonic color and superhydrophobicity in *Morpho* butterfly scales^25^, this multi-purpose optimization is common. Applying a multifunctional design to synthetic systems opens up entirely new technological paradigms that exploit coupling between otherwise distinct phenomena. For example, nanoscale optomechanical crystals have been shown to enable strong phonon-mediated nonlinear optical effects and precise optical measurement of mechanical vibrations through strong interactions between the structure’s photonic and phononic properties^39^. Likewise, optofluidic crystals introduced here enable many new capabilities through strong interactions between fluidic and optical effects. Fluids provide a means to dynamically control and freeze into place tunable degrees of disorder in the 3D photonic structure. In low-refractive-index systems such as the one studied here, this percolation lithography may be used to tune the appearance of structural colors (brightness, hue and diffusivity) in a way that is compatible with large-scale manufacturing. If applied to 3D photonic crystals with higher refractive index contrast, sufficient to produce complete 3D photonic band gaps, additional interesting phenomena such as spontaneous cavity formation and random lasing may be observed. Percolation lithography should also be able to dynamically tune disorder in 1D and 2D planar photonic crystals, creating statistical perturbations that occur over a large scale and can be continually adjusted within the same structure. Conversely, the photonic properties of optofluidic crystals provide a direct optical readout of fluidic behavior (imbibition, evaporation). This readout enables colorimetric analysis of the surface properties^31^ and volatility profile of a liquid or liquid mixture.

## Materials and Methods

### IOF Synthesis

Inverse-opal films (IOFs) were deposited on silicon substrates using a colloidal co-assembly technique described previously^31^,^32^. After fabrication, the surfaces of all IOFs were functionalized with n-decyl trichlorosilane (DEC) as described previously^31^.

### Drying Experiments

Each drying experiment started with immersing a DEC-functionalized IOF in an aliphatic liquid (decane, undecane, dodecane, dodecane-hexadecane and decane-PDMS mixtures were used in different experiments, as indicated in the main text) leading to complete wetting of all pores. Once pores had completely filled (as observed via the color^31^), the IOF was removed and immediately placed under running DI water for 5–10 s. The running DI water, immiscible with the aliphatic liquid and having a poorer affinity for the DEC-functionalized pore surfaces, served to clean the surface of any excess aliphatic liquid. The aliphatic liquid that remained trapped in the pores formed a lubricating thin film that allowed all excess water to slide off^37^,^38^. The IOF was then placed under a modified optical microscope (Leica) that had the capacity to take bright-field (BF), dark-field (DF) and Bertrand-lens (BL) images as well as reflectance spectra (RS)^36^. Subsequent drying experiments were done on the same region of the IOF to capture the full time-evolution in BF, DF, BL, and RS. Dodecane was chosen for the bulk of the characterization experiments because of its volatility, which was low enough to give ample time after DI water flushing had completed to set up the imaging experiment before the pores had begun to empty.

### Partial Wetting Experiments

IOFs, uniformly functionalized with n-decyl trichlorosilane (DEC), were immersed in different mixtures of water and ethanol (Fig. 3A–C). Adjusting the ratio of water and ethanol in the mixtures allowed us to create a continuum of *θ*_c_ values in the pores^31^ and identify the concentration range that induces incomplete wetting of the pores. Partial wetting in DEC-functionalized IOFs was characterized by BF, DF, BL and RS while they were submerged in a small dish containing mixtures of water and ethanol in this concentration range.

### Creating permanent replicas of disordered structures by percolation lithography

#### Drying experiments

Before creating a frozen pattern on an IOF, a complete dodecane drying experiment was run with the time evolution of the RS recorded to use as the comparison reference (see Fig. 7B). PDMS (Sylgard 184, 10:1 base:cross-linker) was prepared and mixed with decane at different ratios. After completing the dodecane experiment, the IOF was submerged in a chosen PDMS-decane solution and then placed under running DI water as in normal drying experiments. The sample was then left at room temperature for 3–5 h, ensuring the volatile component had evaporated, and then placed in an oven at 70 °C overnight to allow the PDMS to cure. Samples were characterized after PDMS curing.

#### Partial wetting

Permanent replicas in the partially infiltrated samples were produced using solvent exchange. To ensure minimal disturbance of the wetting state and consistent miscibility with the epoxy during the exchange, we first exchanged the starting ethanol-water mixture (55–100% ethanol) with a 50:50 ethanol-water mixture and then performed a second exchange with the epoxy resin. The solvent exchange tests (see Fig. 8B,C) were carried out by first imaging the IOF in the starting ethanol-water mixture and then quickly removing the IOF and submerging it in a second dish containing DI water. To freeze partial wetting patterns, we submerged the IOF first in the desired ethanol-water mixture. We then rapidly removed it and submerged it in a 50% ethanol solution to prime it for epoxy exchange. After leaving it to rest for at least 5 min to ensure equilibration in the pores, we removed the IOF from this solution and rapidly submerged it (before drying could occur) in a container with the UV-curable epoxy resin (EPO-TEK OG 142). After ~30 s of immersion in the resin solution with gentle agitation, the IOF was removed from the resin and sandwiched between two thin layers of cured PDMS. This was done to squeeze out most of the excess epoxy on top of the IOF surface and provide more consistent imaging conditions before and after curing. After imaging and spectral characterization of the uncured samples they were then cured under a UV lamp for 30 min, solidifying the partially wet IOF.

### Simulations

Simulations were built based on the model described previously for partial wetting in inverse opals^31^. To facilitate a computationally efficient simulation over as large an area as possible (large areas were needed to adequately capture the statistics of percolation phenomena) with results that were easy to visualize, a 2D IOF lattice (hexagonal) was used. The model generated a lattice with a length of 200 units with lateral periodic boundary conditions and a thickness of 25 layers. Nearest neighbors were connected by necks. Neck angles (*ϕ*_0_) were randomly assigned to each nearest-neighbor connection according to a normal distribution with a mean and standard deviation of 19.6° and 3.2° respectively, matching typical experimental values previously measured^31^. To calculate a partial wetting pattern, we first assigned an intrinsic contact angle (*θ*_c_) for a liquid that was close to the mean neck angle (see Fig. 2A for values used). Then, starting with a completely filled top layer (as these are half-spheres in our IOFs and have no re-entrant curvature to pin the meniscus^31^), the simulation filled all pores with paths of inter-pore fluid connectivity (i.e., where *ϕ*_0_ > *θ*_c_) connecting them to the top filled layer. Drying patterns were simulated from an initial condition of a completely empty top layer (half-pores) with all other pores filled. The simulation then emptied the pores one-by-one, emptying at each step the filled pore associated with the largest neck angle (weakest pinning effect) that connected a filled and empty pore in the current configuration.

For each filling state analyzed (partial wetting or drying), a refractive index map of the structure was created with a spatial resolution of 1/30 of the lattice spacing using a liquid refractive index of 1.35 and a matrix refractive index of 1.4. Fourier transforms (FTs) of these maps were also taken, where the image was first made square by adding a substrate with the matrix refractive index of sufficient thickness below. These maps are shown in Fig. 2A,B. Filling vs. depth maps (Fig. S1) and disorder profiles as a function of total filling fraction (Fig. 2C,D) reflect the average result of 10 successive simulations, where a new random neck distribution is generated for each. This was done to eliminate artifacts arising from particular neck distributions. The disorder parameter (Fig. 2C,D) was calculated from the FTs using the fraction of the total spectral density that lay within the first Brillouin zone, but outside of the vertical axis. We excluded the vertical axis in order to discount effects of the zero-spatial-frequency component at the origin as well as the effects of the top and bottom surfaces of the IOF.

## Additional Information

**How to cite this article**: Burgess, I. B. *et al.* Tuning and Freezing Disorder in Photonic Crystals using Percolation Lithography. *Sci. Rep.* **6**, 19542; doi: 10.1038/srep19542 (2016).

## Supplementary Material

Supplementary Movie M1

Supplementary Movie M2

Supplementary Movie M3

Supplementary Figures
