# Supplementary material for: Tuning and Freezing Disorder in Photonic Crystals using Percolation Lithography
Source: Sci Rep. 2016 Jan 21;6:19542. doi: 10.1038/srep19542 (PMC4726398; doi:10.1038/srep19542)
Supplement: Supplementary Figures [file srep19542-s4.pdf]

Supporting Information for

Tuning and Freezing Disorder in Photonic Crystals using Percolation Lithography

Ian B. Burgess[1,2*], Navid Abedzadeh[3], Theresa M. Kay[2,3], Anna V. Shneidman[4], Derek J. Cranshaw[3], Marko Lončar[3*], Joanna Aizenberg[2,3,4,5*]
[1]*Leslie Dan Faculty of Pharmacy, University of Toronto, Toronto, Ontario Canada*
[2]*Wyss Institute for Biologically Inspired Engineering, Harvard University, Cambridge, MA, USA*
[3]*School of Engineering and Applied Sciences, Harvard University, Cambridge, MA, USA*
[4]*Department of Chemistry and Chemical Biology, Harvard University, Cambridge, MA, USA*
[5]*Kavli Institute for Bionano Sciences and Technology, Harvard University, Cambridge, MA, USA*
**email: ib.burgess@utoronto.ca, loncar@seas.harvard.edu, jaiz@seas.harvard.edu*

Supporting Figures

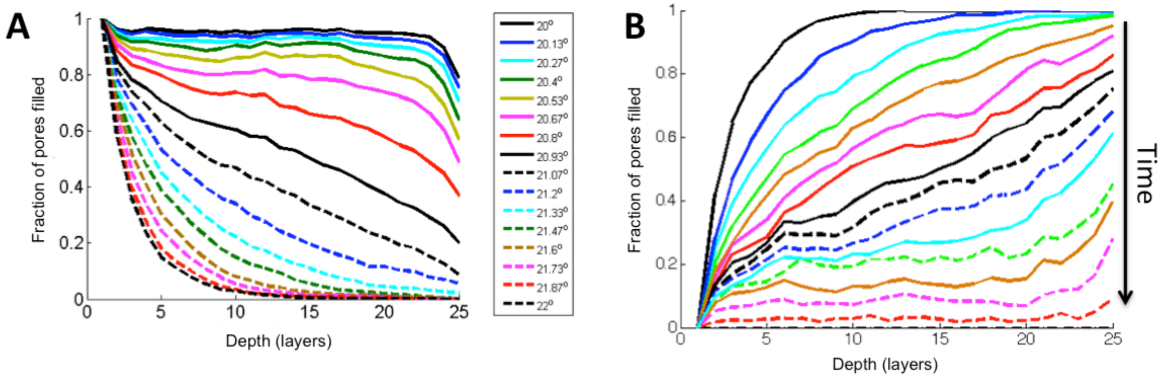

Figure S1. Simulated filling depth profiles during partial wetting and drying. Fraction of filled pores at each layer evolving as a function of θ_c during partial wetting (A) and as a time progression during drying (B).

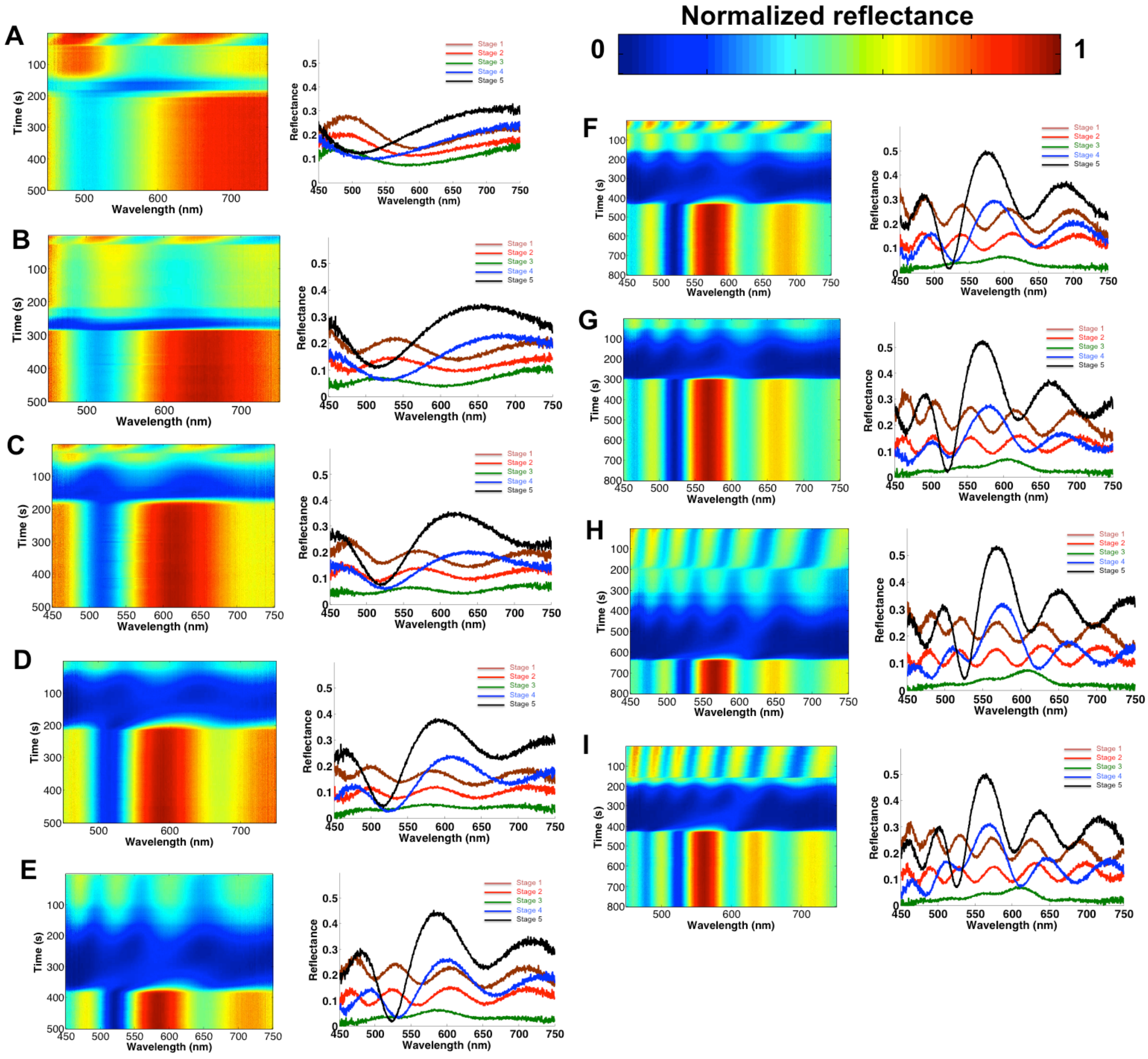

Fig. S2. Time evolution of reflectance at normal incidence during drying of dodecane (left) and corresponding spectra at the five stages defined by total reflectance (right). A – 3-layer IOF; B – 4-layer IOF; C – 5-layer IOF; D – 6-layer IOF; E – 7-layer IOF; F – 8-layer IOF; G – 9-layer IOF; H – 10-layer IOF; I – 11-layer IOF;

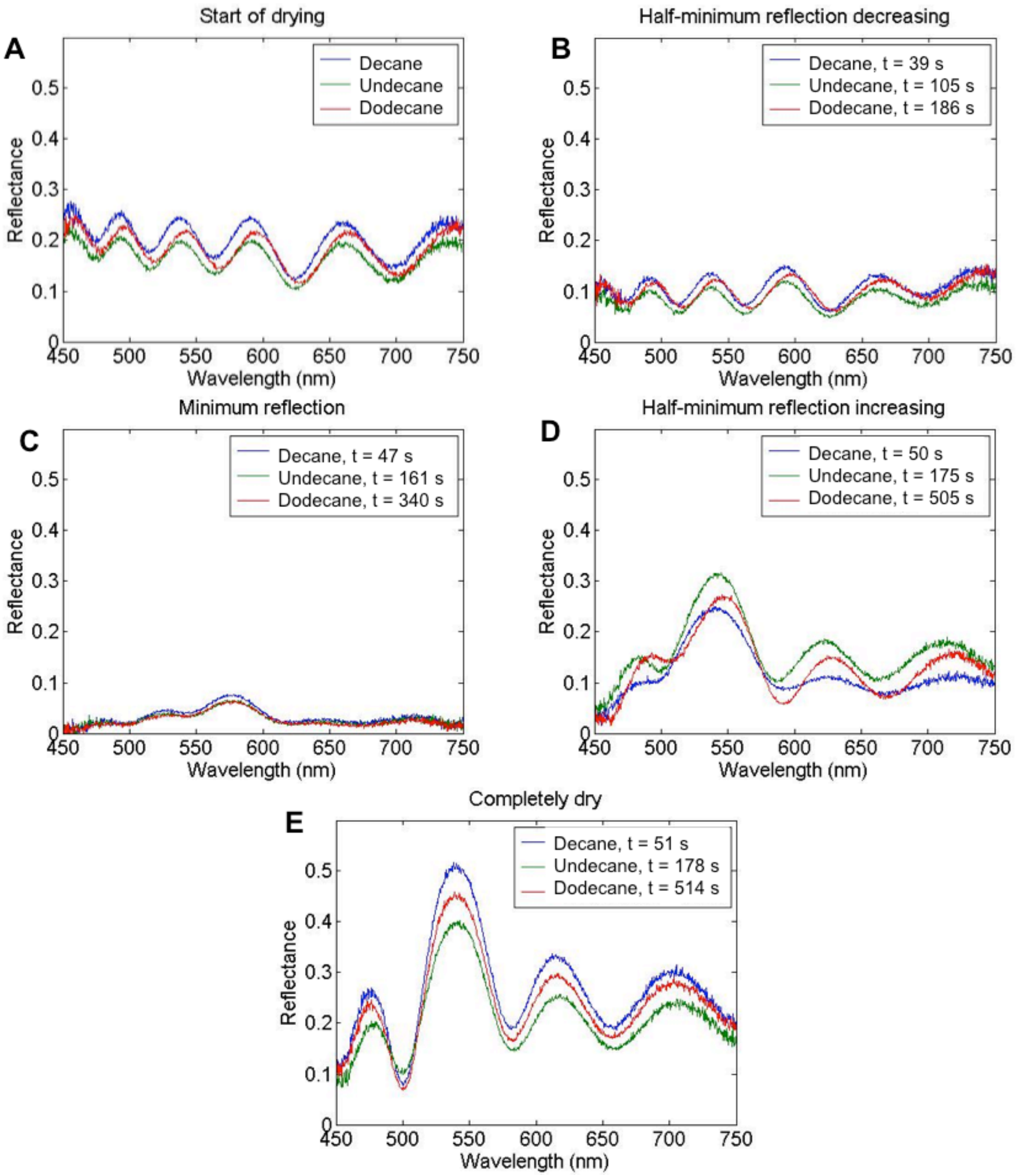

Figure S3. Drying sequence in different liquids. Comparison of normal-incidence reflection spectra of an IOF (9 layers) at the five stages of drying: (A) Stage 1: start of drying; (B) Stage 2: total reflectance decreases to half of the minimum with respect to stage 1; (C) Stage 3: point of minimum total reflectance; (D) Total reflectance recovered to halfway between the minimum and that of a dry film; (E) Stage 5: dry film.

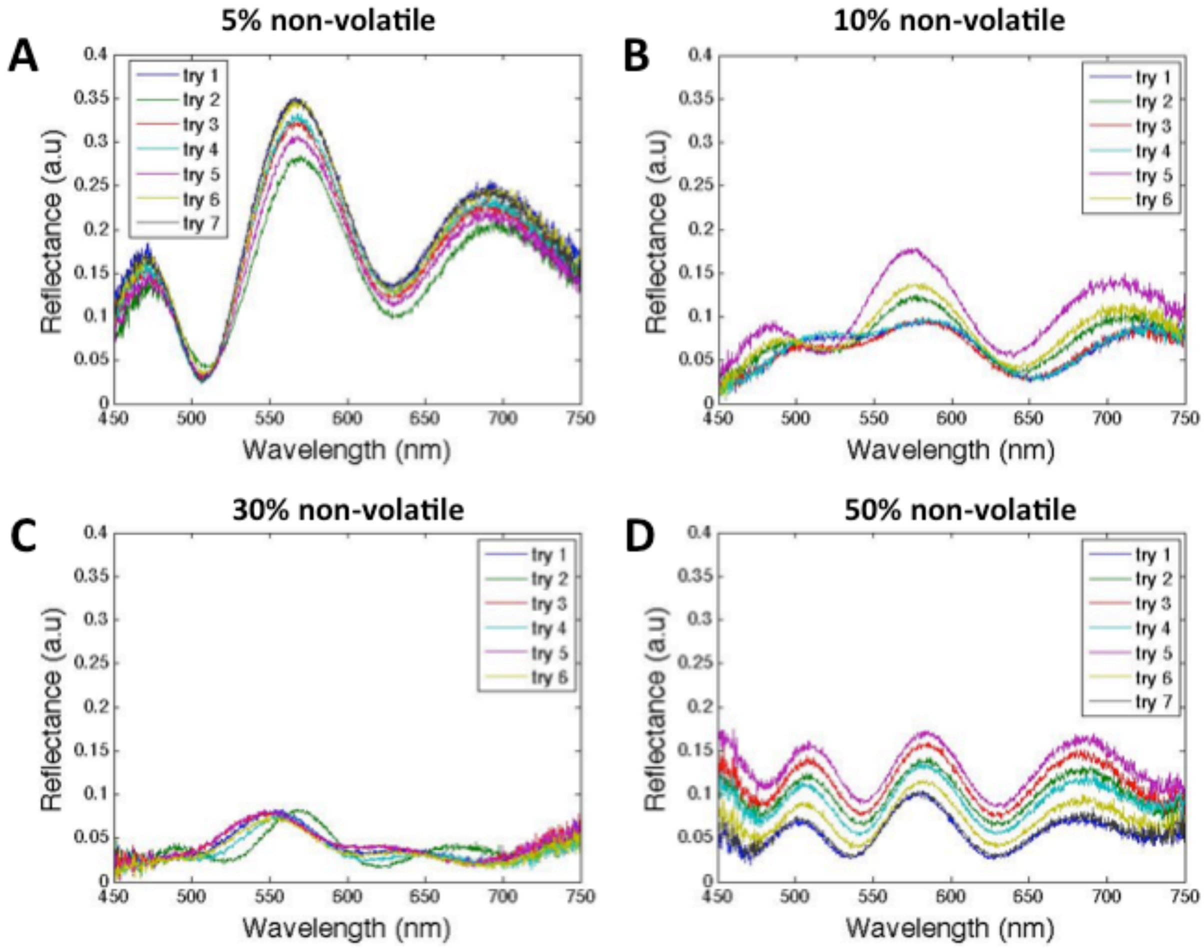

Figure S4. Detecting volatile fraction from disorder. Final reflectance spectra (showing several repeats) after evaporation of dodecane from dodecane-hexadecane mixtures with hexadecane volume fractions of 5% (A), 10% (B), 30% (C), and 50% (D).

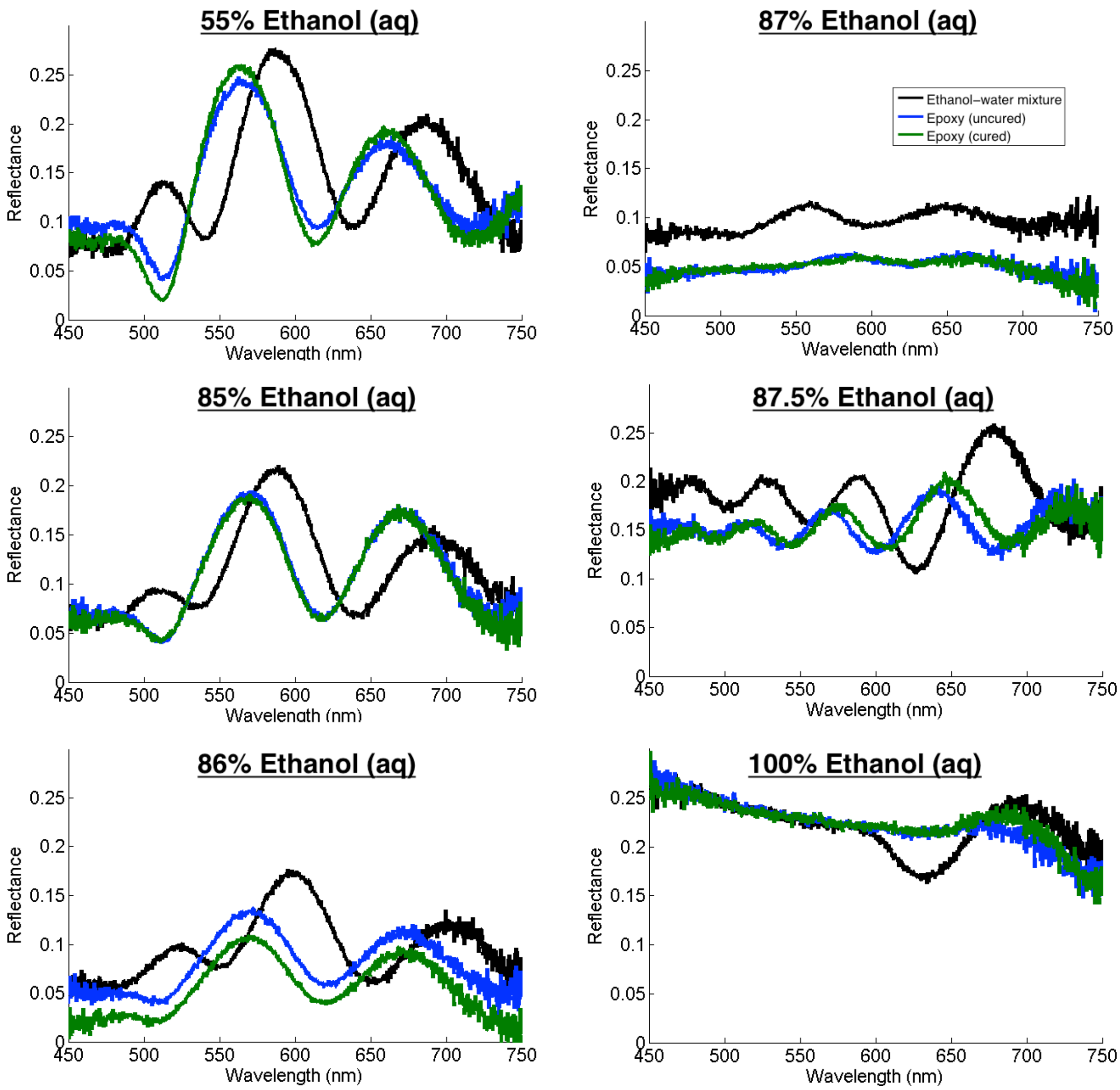

Figure S5. Freezing of partial wetting via epoxy resin exchange. Reflectance spectra at normal incidence taken of IOFs (9-layers) after immersion in an ethanol water-mixture (black curves), after exchange of the mixture with epoxy resin (blue curves) and then after the epoxy resin (OG 142) has been cured (green curves).
